# Unlocking potential: language mindsets, psychological well-being, and English achievement among Chinese higher vocational students

**DOI:** 10.3389/fpsyg.2026.1969912

**Published:** 2026-10-05

**Authors:** Qian Wei

**Affiliations:** Department of Basic Education, Shanghai Zhongqiao Vocational and Technical University, Shanghai, China

**Keywords:** College English Test Band 4, English achievement, fixed language mindset, growth language mindset, higher vocational students, PERMA, positive psychology, psychological well-being

## Abstract

This cross-sectional study examined how language mindset beliefs are associated with psychological well-being and English achievement among Chinese higher vocational students, an underrepresented population in educational psychology. A total of 1,030 students from a private vocational and technical university completed the PERMA-Profiler and Language Mindset Inventory and reported their latest College English Test Band 4 (CET-4) score. Spearman rank-order correlations, regression-based moderation analysis with heteroscedasticity-robust standard errors, Mann–Whitney *U* tests, and Kruskal–Wallis *H* tests were used. Growth language mindset (GLM) was strongly associated with overall psychological well-being (*r_s_* = 0.589), with its strongest observed PERMA subscale associations involving meaning (*r_s_* = 0.583), accomplishment (*r_s_* = 0.568), and engagement (*r_s_* = 0.542). Fixed language mindset (FLM) showed weaker associations with positive PERMA subscale scores and its strongest association was with negative emotion (*r_s_* = 0.408). CET-4 scores were only weakly associated with well-being (*r_s_* = 0.110), GLM (*r_s_* = 0.127), and FLM (*r_s_* = −0.080). Neither the well-being × GLM nor the well-being × FLM interaction was significant, indicating no evidence that either language mindset factor moderated the association between well-being and CET-4 achievement. Demographic differences in CET-4 achievement by gender, only-child status, and hometown locale were statistically significant but small; male students also reported slightly higher FLM scores than female students. These findings suggest that the two theory-grounded LMI composite scores are more closely related to learners’ psychological functioning than to standardized English achievement, although their current-sample structural distinctiveness should be interpreted cautiously.

## 1 Introduction

Moving beyond the traditional focus on psychological deficits and disorders, positive psychology (PP) emphasizes the study of human strengths, optimal functioning, and pathways toward flourishing (Seligman and Csikszentmihalyi, 2000; Sheldon and King, 2001). Within educational contexts, this perspective shifts attention from merely correcting learning difficulties toward understanding the psychological resources that enable students to persist, engage, and thrive.

Building on authentic happiness theory, Seligman (2012) proposed Well-Being Theory (PERMA), comprising five measurable elements: positive emotion, engagement, relationships, meaning, and accomplishment. Drawing on positive psychology, second-language (L2) researchers have increasingly examined how learners’ motivation and engagement are associated with both positive and negative emotional experiences (Dewaele et al., 2019; Dewaele and MacIntyre, 2014; MacIntyre et al., 2019).

In parallel, individuals’ self-evaluations influence their beliefs, feelings, and actions (Dweck, 2000). Mindset theory distinguishes growth mindsets, in which ability is viewed as developable, from fixed mindsets, in which ability is viewed as relatively stable (Dweck, 2000, 2007; Dweck and Leggett, 1988). Extending this framework, language mindset research distinguishes fixed language mindset (FLM) from growth language mindset (GLM) (Mercer and Ryan, 2009). Recent L2 research has further conceptualized language mindsets as domain-specific beliefs concerning whether language ability can be developed (Lou and Noels, 2016, 2019).

Previous studies have linked growth mindset with better mental-health outcomes among Chinese university students (Tao et al., 2022) and with English-language performance through motivational-affective variables among Chinese EFL university students (Hu et al., 2022). However, limited research has examined how language-specific mindsets are related to multidimensional well-being among English learners in higher vocational education contexts.

In China, higher vocational students (HVSs) represent a substantial yet underexamined population in educational psychology. Compared with students in academically oriented universities, HVS learners may encounter distinctive educational trajectories, diverse academic backgrounds, and contextual pressures related to future employment opportunities (Ling et al., 2023; Xue and Li, 2022). However, existing discussions of higher vocational learners have frequently adopted deficit-oriented perspectives, emphasizing academic limitations rather than psychological resources and developmental potential.

From a PP perspective, examining students’ well-being and language mindsets may provide a more comprehensive understanding of how HVSs experience English learning. Therefore, investigating the relationships among well-being, language mindsets, and English achievement may contribute to a more strengths-based understanding of learner development.

The current study contributes to educational psychology by extending language mindset research beyond achievement outcomes and examining its association with psychological well-being among an underrepresented learner population.

Practically, the findings may inform psychologically supportive policy and pedagogy in Chinese higher vocational education while avoiding the assumption that psychological factors alone determine standardized achievement.

Against this theoretical background, the present study adopts a PP perspective to examine language learning among Chinese HVSs. Specifically, this study investigates whether language-specific beliefs are associated with students’ general psychological well-being and English achievement, while also considering demographic and educational background differences.

By focusing on HVSs, this study aims to extend educational psychology research beyond traditionally examined university populations and provide a strengths-based understanding of learner development in English education contexts.

The study addresses the following research questions:

*RQ1*: What are the associations among language mindsets, psychological well-being, and English achievement among Chinese HVSs?

*RQ2*: How are specific dimensions of psychological well-being associated with growth and fixed language mindsets among Chinese HVSs?

*RQ3*: Does the association between psychological well-being and English achievement vary according to students’ growth and fixed language mindsets?

*RQ4*: How do demographic and learning-history characteristics relate to differences in language mindsets, psychological well-being, and English achievement among Chinese HVSs?

## 2 Literature review

### 2.1 Positive psychology and psychological well-being

Positive psychology (PP) shifts psychological inquiry from the treatment of deficits toward the study of human strengths, optimal functioning, and flourishing (Seligman and Csikszentmihalyi, 2000; Sheldon and King, 2001). Within educational contexts, this perspective highlights that successful learning involves not only academic achievement but also learners’ psychological functioning, including engagement, meaning, positive emotions, and accomplishment (Seligman, 2012; Seligman et al., 2009).

Building on authentic happiness theory, Seligman (2012) proposed Well-Being Theory (PERMA), comprising five measurable elements: positive emotion, engagement, relationships, meaning, and accomplishment. Drawing on positive psychology, second-language (L2) researchers have increasingly examined how learners’ motivation and engagement are associated with both positive and negative emotional experiences (Dewaele et al., 2019; Dewaele and MacIntyre, 2014; MacIntyre et al., 2019). Importantly, well-being research in second language education has increasingly moved beyond examining only positive emotions and has begun to recognize the coexistence of positive and negative emotional experiences (MacIntyre et al., 2019). This literature concerns language-learning experiences; in the present study, the PERMA-Profiler measures general psychological well-being rather than EFL-specific well-being or classroom emotion. Cross-cultural research suggests that positive and negative affect need not operate as mutually exclusive states. Some East Asian samples have shown greater co-occurrence of pleasant and unpleasant emotions, a pattern discussed in relation to dialectical thinking and emotional complexity (Schimmack et al., 2002; Spencer-Rodgers et al., 2010; Grossmann et al., 2016). This literature provides a possible interpretive context for examining negative emotion alongside positive well-being dimensions; it does not establish a cultural mechanism in the present sample.

More recent China-specific evidence indicates that culturally patterned thinking styles may be associated with psychological functioning in differentiated ways. Huang et al. (2024) found that the connectedness dimension of Chinese thinking style was positively associated with mental health, whereas the contradiction dimension showed a negative association; psychological resilience statistically mediated these relationships. These findings suggest that Chinese thinking styles are multidimensional rather than having uniformly positive or negative psychological consequences, but they do not explain the present data because these constructs were not measured.

### 2.2 Language mindsets as domain-specific psychological beliefs

Mindset theory proposes that individuals differ in their beliefs regarding whether personal abilities are fixed or developable. Individuals holding fixed mindsets perceive ability as relatively stable, whereas those endorsing growth mindsets view ability as malleable through effort and learning (Dweck, 2007; Dweck and Leggett, 1988).

Mindsets may operate in domain-specific ways rather than as uniform general traits (Dweck et al., 1995). Extending mindset theory to second-language learning, Mercer and Ryan (2009) and Lou and Noels (2016, 2019) conceptualized language mindsets as learners’ beliefs about the development of language ability. GLM reflects the belief that language competence can be improved through effort and effective strategies, whereas FLM reflects the belief that language ability is relatively stable. Lou and Noels (2017) operationalized language mindsets through three belief domains: general language intelligence beliefs (GLB), second-language aptitude beliefs (L2B), and age-sensitivity beliefs (ASB), each represented by growth- and fixed-oriented items. At the higher-order mindset level, the LMI is regarded as a two-factor model comprising growth/incremental and fixed/entity orientations. Each factor is expressed across the same three belief domains (GLB, L2B, and ASB), so GLM and FLM are conceptually related but separately scored dimensions rather than opposite endpoints.

Compared with general academic domains, foreign language learning provides a unique context for examining mindset beliefs because language development often requires sustained effort, tolerance of ambiguity, and continuous exposure to challenges (Wang et al., 2021; Xu and Ba, 2022). Therefore, language mindsets may influence not only achievement-related behaviors but also learners’ psychological experiences during the learning process.

### 2.3 Language mindsets and psychological well-being

Growth mindset has been linked to school engagement and psychological well-being among Chinese students (Zeng et al., 2016). Among Chinese university students, Jia et al. (2022) further found that growth mindset was positively associated with happiness and negatively associated with anxiety, with perceived stress and psychological resilience statistically mediating these relationships. In L2 contexts, growth-oriented language beliefs have also been associated with engagement and motivational resources (Sadoughi et al., 2023; Zhong et al., 2024).

Among Chinese university EFL learners, Hu et al. (2022) reported associations between growth mindset, English performance, grit, and foreign-language enjoyment, while Zhong et al. (2024) found positive relationships among growth language mindset, positive emotions, and engagement. Together, this work suggests motivational-affective pathways linking mindset beliefs to learning experiences, but it does not directly establish how growth and fixed language mindsets relate to multidimensional well-being among higher vocational learners.

Although previous research has established relationships between growth mindsets and academic outcomes, less attention has been paid to their associations with psychological well-being, especially in language learning contexts. Growth-oriented beliefs may support well-being by encouraging learners to interpret difficulties as temporary challenges rather than evidence of limited ability. Such interpretations may foster persistence, engagement, and a stronger sense of meaning.

This possibility is consistent with the language mindset–meaning system proposed by Lou and Noels (2019), which situates language mindsets within broader processes of meaning-making, attribution, effort beliefs, goals, self-regulation, and emotional experience. From this perspective, growth-oriented language beliefs may be relevant to how learners interpret challenges and construct purpose during language learning. Fixed language mindsets may likewise be associated with negative emotional experiences when academic difficulties are interpreted as indicators of insufficient ability. These are theoretical possibilities; the present cross-sectional data test associations rather than these explanatory processes.

### 2.4 Language mindsets as exploratory moderators

The preceding literature supports possible main-effect and correlational links among language mindset beliefs, well-being, and achievement, but it does not establish that mindset must change the strength of the well-being–achievement association. The interaction model was therefore included as an exploratory extension of the language mindset–meaning system: when learners experience greater well-being, growth-oriented beliefs may plausibly be associated with greater persistence and use of learning opportunities, whereas fixed-oriented beliefs may be associated with less adaptive responses to challenge. These are reasons to examine moderation, not confirmatory directional predictions. A correlation of either mindset with well-being or CET-4 would not, by itself, constitute evidence of moderation.

### 2.5 English achievement in Chinese higher education

In the Chinese higher education context, College English Test Band 4 (CET-4) is commonly used to assess undergraduate students’ English proficiency and remains influential in academic and employment contexts (Zheng and Cheng, 2008). CET-4 provides a standardized external indicator of English achievement, reflecting multiple factors beyond psychological characteristics, including prior language exposure, learning opportunities, and test preparation (Bai, 2020; Ma, 2025), although the scores were self-reported in the present study. Therefore, examining CET-4 alongside psychological variables may provide a more comprehensive understanding of English learning outcomes.

CET-4 scores should also be interpreted with attention to the conventions surrounding the examination. Although 425 is widely used in practice as a reference point for CET-related progression and eligibility, CET-4 does not have an officially designated pass line (Xu and Liu, 2019; Yao et al., 2023). Accordingly, the present study treats CET-4 as a continuous indicator of English achievement rather than as a binary pass/fail outcome.

### 2.6 Higher vocational students as an underrepresented learner population

Higher Vocational Students (HVSs) represent an important yet understudied population in educational psychology. Compared with students enrolled in academically oriented universities, vocational students often have diverse educational backgrounds and learning trajectories (Ling et al., 2023; Xue and Li, 2022).

Rather than viewing higher vocational learners through deficit-based perspectives, a positive psychology approach highlights their psychological resources, developmental potential, and capacity for growth. Despite growing interest in mindset and positive psychology, three limitations remain in the existing literature. First, mindset research has predominantly emphasized achievement-related outcomes. Second, comparatively little research has examined associations between language-specific mindsets and general multidimensional psychological well-being. Third, Chinese HVSs remain underrepresented in educational psychology and L2 research. Addressing these gaps, the present study examines cross-sectional associations among language mindsets, general psychological well-being, English achievement, and learner-background characteristics in this population.

## 3 Materials and methods

### 3.1 Research design

A quantitative cross-sectional research design was employed to examine associations among language mindsets, general psychological well-being, English achievement, and demographic characteristics among Chinese HVSs. Grounded in positive psychology and language mindset theory, the study explored how general psychological well-being and language-specific beliefs were associated in an English as a foreign language (EFL) learning context; it did not measure EFL-specific well-being or classroom-specific emotional experience.

Specifically, the study investigated (a) the associations among language mindsets, psychological well-being, and CET-4 achievement; (b) the relationships between specific dimensions of psychological well-being and language mindset beliefs; (c) as an exploratory analysis, whether the association between psychological well-being and CET-4 achievement varied as a function of GLM and FLM; and (d) whether students from different demographic and learning-history backgrounds demonstrated differences in language mindsets, psychological well-being, and English achievement.

The following research questions guided the study:

*RQ1*: What are the associations among language mindsets, psychological well-being, and English achievement (CET-4 scores) among Chinese HVSs?

*RQ2*: How are specific dimensions of psychological well-being associated with growth and fixed language mindset beliefs among Chinese HVSs?

*Exploratory RQ3*: Does the association between psychological well-being and English achievement (CET-4 scores) vary according to students’ growth and fixed language mindset beliefs?

*RQ4*: How do demographic and learning-history characteristics relate to differences in language mindsets, psychological well-being, and English achievement among Chinese HVSs?

### 3.2 Participants

Participants were recruited from a private vocational and technical university in Shanghai, China. Students across academic years were invited to participate in the online survey through counselors in their respective departments. Of the 2,206 students approached, 248 declined participation, resulting in 1,958 respondents and a response rate of 88.76%.

Participant characteristics for the final analytic sample are presented in Table 1.

**Table 1:** Participant characteristics.

Variables	Description	Frequency	%
Year of Study	First	340	33
	Second	575	55.8
	Third	105	10.2
	Four or more	10	1
Gender	Female	683	66.3
	Male	347	33.7
Hometown Locales	Rural	339	32.9
	Suburban	314	30.5
	Urban	377	36.6
Only Child	Only Child	558	54.2
	Not Only Child	472	45.8
Accommodation	On-campus	990	96.1
	Commuter	40	3.9
Future Degree	Bachelor	451	43.8
	Top-up	52	5
	Associate	527	51.2
Number of CET-4 Attempts	1st	734	71.3
	2nd	230	22.3
	3rd	48	4.7
	4th or more	18	1.7

*N* = 1,030.

Because CET-4 achievement constituted one of the principal variables in the present study, self-reported CET-4 scores were screened according to the prespecified admissible range of 220–710. Of the 1,958 consenting respondents, 1,030 reported CET-4 scores within this range and were retained as the complete-case analytical sample. The remaining 928 respondents reported CET-4 values outside the specified range and were excluded from subsequent analyses.

The final sample consisted of 683 female students (66.3%) and 347 male students (33.7%). Participants represented different years of study, hometown locales, family structures, accommodation arrangements, future degree pathways, and CET-4 examination histories. Specifically, 339 students were from rural areas, 314 from suburban areas, and 377 from urban areas; 558 were only children and 472 were not; and 990 students lived on campus. The final analytical sample therefore comprised *N* = 1,030 students.

### 3.3 Instruments

#### 3.3.1 PERMA-profiler

The PERMA-Profiler developed by Butler and Kern (2016) was used to assess students’ general psychological well-being based on Seligman’s PERMA framework. It contains five core dimensions: positive emotion, engagement, relationships, meaning, and accomplishment. Overall Well-being was calculated as the mean of the 15 core PERMA items plus the single Overall Happiness item (16 items in total). Negative Emotion, Health, and Loneliness were scored separately and were not included in the Overall Well-being composite. The PERMA-Profiler uses an 11-point response format ranging from 0 (the lowest degree) to 10 (the highest degree) and has demonstrated satisfactory psychometric properties across contexts. A recent Chinese validation supported its reliability and structural validity among Chinese adults (Butler and Kern, 2016; Nie et al., 2024).

#### 3.3.2 Language mindset inventory

The Language Mindset Inventory (LMI) developed by Lou and Noels (2017) was used to assess learners’ beliefs regarding the malleability of language ability. The instrument contains Fixed- and Growth-oriented items across three language-belief domains: general language intelligence beliefs (GLB), second-language aptitude beliefs (L2B), and age-sensitivity beliefs (ASB). Theory-grounded observed composite scores were calculated separately for growth language mindset (GLM) and fixed language mindset (FLM). The instrument consists of 18 items measured on a six-point Likert scale ranging from strongly disagree to strongly agree. Nine items represent GLM and nine represent FLM, with three growth-oriented and three fixed-oriented items in each of the GLB, L2B, and ASB domains. Previous confirmatory-factor analyses have supported the distinction between incremental (growth) and entity (fixed) orientations in the LMI, and the Chinese version has also demonstrated acceptable reliability and structural validity among EFL learners (Lou and Noels, 2017; Yao et al., 2021). In the present study, GLM and FLM were therefore retained as separate continuous factors rather than collapsed into a single composite mindset score.

### 3.4 Procedure and data analysis

#### 3.4.1 Data collection procedure

The doctoral-dissertation research received approval from the Chapman University Institutional Review Board (IRB-23-298; approved May 24, 2023). Before local data collection, approval/authorization was also obtained from the Human Research Ethics Committee of the participating university. These approvals served the doctoral research oversight and local institutional roles, respectively. Departmental counselors circulated the online survey link through WeChat; participation was voluntary, and students reviewed study information and provided electronic informed consent before answering. Of the 2,206 students approached, 248 declined participation. Responses were anonymous, treated confidentially, and used solely for research purposes. Data collection lasted 4 weeks.

#### 3.4.2 Data preparation and reliability analysis

Before statistical analyses, data screening procedures were conducted. Because CET-4 scores were self-reported, responses outside the official score range (220–710) were excluded from analysis. After data cleaning, 1,030 valid cases were retained.

Internal consistency was evaluated using Cronbach’s alpha for the same 1,030 participants included in the main analyses. The 16-item Overall Well-being composite (15 core PERMA items plus Overall Happiness) demonstrated excellent internal consistency (*α* = 0.970). Reliability coefficients for the separately scored indicators were also satisfactory: positive emotion (*α* = 0.885), engagement (*α* = 0.817), relationships (*α* = 0.855), meaning (*α* = 0.890), accomplishment (*α* = 0.898), health (*α* = 0.911), and negative emotion (*α* = 0.830). The full 23-item PERMA-Profiler yielded α = 0.960; this coefficient is reported as scale-wide internal consistency and is not evidence that all 23 items represent a single construct.

The language mindset measures similarly demonstrated high internal consistency. The nine-item GLM measure yielded α = 0.950 and the nine-item FLM measure yielded *α* = 0.936. At the belief level, coefficients were 0.952 for growth GLB, 0.910 for growth L2B, 0.946 for growth ASB, 0.888 for fixed GLB, 0.842 for fixed L2B, and 0.903 for fixed ASB. The complete 18-item LMI yielded *α* = 0.930. Overall, all multi-item measures exceeded the conventional 0.80 criterion for good internal consistency (DeVellis, 2016).

For the LMI, the simple item-level two-factor GLM/FLM WLSMV model fit poorly. A theoretically specified hierarchical WLSMV model with six domain-by-orientation first-order factors and correlated higher-order GLM and FLM factors fit substantially better, although fit remained mixed (CFI = 0.931, TLI = 0.918, RMSEA = 0.108, SRMR = 0.074). The higher-order factors were identifiable and moderately correlated (*r* = 0.451). GLM and FLM were therefore retained as theory-grounded observed composite scores, but their structural distinctiveness in this sample should be interpreted cautiously.

Current-sample CFA results were evaluated alongside internal consistency. For the 15 core PERMA items, the five-factor robust MLR model showed strong standardized item loadings and within-factor convergence, but overall fit was poor. Latent factor correlations were extremely high and some exceeded 1.00; discriminant validity among the five core PERMA dimensions was therefore not established. Accordingly, the dimension-level findings are interpreted as observed PERMA subscale patterns rather than evidence of fully distinct latent dimensions.

#### 3.4.3 Statistical analysis

Spearman correlation analyses and non-parametric group comparisons were conducted using SPSS Version 29. The additional moderation analysis for RQ3 was estimated by Python 3.13 and statsmodels 0.14.6. Because preliminary analyses indicated that the continuous variables deviated from normal distributions, non-parametric procedures were retained for correlation and group-comparison analyses, while HC3 heteroscedasticity-robust standard errors were used for regression-based moderation inference.

For RQ1 and RQ2, Spearman rank-order correlation analyses were conducted to examine associations among language mindsets, observed PERMA subscale scores, and CET-4 achievement. RQ3 was exploratory. The model was examined because language-mindset beliefs may plausibly shape how psychological resources are translated into sustained language-learning behavior and achievement; however, prior associations of mindset, well-being, and CET-4 scores do not by themselves imply an interaction. The analysis therefore retained theory-grounded GLM and FLM observed composite scores as separate continuous predictors and tested, without a confirmatory directional prediction, whether either conditioned the well-being–achievement association. Overall well-being, GLM, and FLM were mean-centered before forming the well-being × GLM and well-being × FLM interaction terms, and the two mindset composite scores were entered simultaneously. HC3 heteroscedasticity-robust standard errors and 95% confidence intervals were used for regression inference. A statistically significant interaction term was interpreted as evidence that the well-being–achievement association varied according to the corresponding language mindset composite. For RQ4, demographic differences were examined using Mann–Whitney *U* tests and Kruskal–Wallis *H* tests. Mann–Whitney *U* tests were applied for comparisons involving two-group variables (gender, accommodation status, and only-child status), whereas Kruskal–Wallis *H* tests were used for variables involving three or more groups (year of study, hometown locale, future educational plans, and number of CET-4 attempts).

For RQ1 and RQ2, a revision-stage Benjamini–Hochberg false-discovery-rate sensitivity analysis was applied separately within the six planned RQ1 correlations and the 64 RQ2 correlations. When significant RQ4 group differences were identified, *post hoc* pairwise comparisons were conducted with Bonferroni adjustment within the relevant omnibus test. Effect sizes for Mann–Whitney *U* tests were expressed as *r*, whereas effect sizes for Kruskal–Wallis *H* tests were estimated using *η^2^_H_ = (H – k + 1)/(N – k)*, where *H* is the Kruskal–Wallis statistic, *k* is the number of groups, and *N* is the total sample size (Pallant, 2016). To make the scope of RQ4 transparent, the complete 17-outcome × 7-group comparison matrix is reported in Supplementary Table S2. As a sensitivity analysis, false-discovery-rate adjustment was applied separately to the 28 primary RQ4 tests involving CET-4, Overall Well-being, GLM, and FLM, and to the complete 119-test RQ4 matrix. These revision-stage sensitivity analyses do not replace the planned tests or the within-omnibus Bonferroni *post hoc* adjustment.

## 4 Results

### 4.1 Descriptive statistics

The final sample consisted of 1,030 Chinese higher vocational students, including 683 female students (66.3%) and 347 male students (33.7%). Participants represented diverse demographic backgrounds, including rural, suburban, and urban hometown locales, different academic years, family structures, and CET-4 examination experiences.

Descriptive analyses showed that participants obtained an average CET-4 score of 413.48 (*SD* = 77.58), which was slightly below the commonly referenced benchmark of 425. The overall psychological well-being score was *M* = 7.02 (*SD* = 1.79) on the original 0–10 metric, while GLM scores were higher than FLM scores (*M* = 4.43 vs. *M* = 3.85).

Descriptive statistics for the main study variables and their dimensions are presented in Table 2. Participants obtained a mean CET-4 score of 413.48 (*SD* = 77.58). Overall psychological well-being was *M* = 7.02 (*SD* = 1.79) on the original 0–10 metric. Relationships showed the highest observed mean among the positive PERMA subscales (*M* = 7.13, *SD* = 1.89), and accomplishment the lowest (*M* = 6.87, *SD* = 1.96). Participants also reported a mean Negative Emotion score of 5.92 (*SD* = 2.15). Regarding language mindsets, the mean GLM score (*M* = 4.43, *SD* = 0.92) was higher than the mean FLM score (*M* = 3.85, *SD* = 1.11). Among the growth-oriented beliefs, growth ASB showed the highest mean (*M* = 4.54, *SD* = 1.02), whereas fixed L2B had the highest mean among the fixed-oriented dimensions (*M* = 3.99, *SD* = 1.15).

**Table 2:** Descriptive statistics and internal consistency of English achievement, psychological well-being, and language mindsets.

Construct	Variable/Dimension	*M*	SD	Mdn	IQR	Min–Max	*α*
English achievement	CET-4	413.48	77.58	415.00	75.00	220–710	–
Psychological well-being	Overall well-being	7.02	1.79	6.94	2.25	0–10	**0.970**
	Positive emotion	7.11	1.93	7.00	2.67	0–10	0.885
	Engagement	6.94	1.83	7.00	2.33	0–10	0.817
	Relationships	7.13	1.89	7.00	2.33	0–10	0.855
	Meaning	6.99	2.01	7.00	2.67	0–10	0.890
	Accomplishment	6.87	1.96	6.67	2.33	0–10	0.898
	Health	7.07	2.06	7.00	3.00	0–10	0.911
	Negative emotion	5.92	2.15	5.67	2.33	0–10	0.830
Fixed language mindset	Overall FLM	3.85	1.11	3.89	1.42	1–6	**0.936**
	Fixed GLB	3.75	1.30	4.00	1.67	1–6	0.888
	Fixed L2B	3.99	1.15	4.00	1.67	1–6	0.842
	Fixed ASB	3.82	1.25	4.00	1.67	1–6	0.903
Growth language mindset	Overall GLM	4.43	0.92	4.33	1.00	1–6	**0.950**
	Growth GLB	4.38	1.06	4.17	1.00	1–6	0.952
	Growth L2B	4.38	1.01	4.17	1.00	1–6	0.910
	Growth ASB	4.54	1.02	4.67	1.00	1–6	0.946

*N* = 1,030. M, mean; SD, standard deviation; Mdn, median; IQR, interquartile range (Q3 − Q1); α, Cronbach’s alpha; CET-4, College English Test Band 4; GLM, growth language mindset; FLM, fixed language mindset; GLB, general language intelligence beliefs; L2B, second-language aptitude beliefs; ASB, age-sensitivity beliefs. PERMA descriptive values are reported on the original 0–10 metric. Bold values indicate Cronbach’s alpha coefficients for the three overall composite measures: Overall Well-being, Overall FLM, and Overall GLM.

### 4.2 Associations among language mindsets, psychological well-being, and English achievement (RQ1)

Spearman rank-order correlations were conducted to examine the associations among language mindsets, psychological well-being, and CET-4 achievement. Results are presented in Table 3.

**Table 3:** Spearman correlations among English achievement, psychological well-being, and language mindsets.

Variable	1	2	3	4
1. CET-4	–			
2. Psychological well-being	0.110**	–		
3. FLM	−0.080**	0.182**	–	
4. GLM	0.127**	0.589**	0.244**	–

*N* = 1,030. Values are Spearman rank-order correlation coefficients (*r*_s_). CET-4, College English Test Band 4; FLM, fixed language mindset; GLM, growth language mindset. **p < 0.01 (two-tailed).

As shown in Table 3, GLM demonstrated a strong positive association with psychological well-being (*r_s_* = 0.589, *p* < 0.01), whereas FLM showed a considerably weaker positive association with well-being (*r_s_* = 0.182, *p* < 0.01). CET-4 scores were weakly positively associated with psychological well-being (*r_s_* = 0.110, *p* < 0.01) and GLM (*r_s_* = 0.127, *p* < 0.01), while a weak negative association was observed between CET-4 scores and FLM (*r_s_* = −0.080, *p* < 0.01). Overall, the strongest association observed in RQ1 was between GLM and psychological well-being.

### 4.3 Associations between psychological well-being dimensions and language mindsets (RQ2)

To address RQ2, Spearman rank-order correlations were examined between language mindset measures and the overall and dimension-specific indicators of psychological well-being. Table 4 presents the correlation patterns for overall growth and fixed language mindsets as well as the corresponding general language intelligence beliefs (GLB), second-language aptitude beliefs (L2B), and age-sensitivity beliefs (ASB).

**Table 4:** Spearman correlations between language mindset measures and psychological well-being.

Language mindset measure	Overall well-being	PE	Engagement	Relationships	Meaning	Accomplishment	Health	NE
Overall GLM	0.589	0.540	0.542	0.525	0.583	0.568	0.482	0.248
Growth GLB	0.580	0.531	0.531	0.502	0.572	0.580	0.503	0.222
Growth L2B	0.561	0.510	0.532	0.493	0.563	0.542	0.463	0.249
Growth ASB	0.479	0.454	0.443	0.448	0.469	0.442	0.374	0.243
Overall FLM	0.182	0.157	0.203	0.165	0.173	0.191	0.158	0.408
Fixed GLB	0.177	0.157	0.182	0.162	0.174	0.187	0.160	0.380
Fixed L2B	0.220	0.196	0.245	0.217	0.198	0.217	0.193	0.397
Fixed ASB	0.189	0.163	0.212	0.154	0.187	0.204	0.168	0.370

*N* = 1,030. Values are Spearman rank-order correlation coefficients (*r*_s_). All correlations were significant at *p* < 0.01 (two-tailed). GLM, growth language mindset; FLM, fixed language mindset; GLB, general language intelligence beliefs; L2B, second-language aptitude beliefs; ASB, age-sensitivity beliefs; PE, positive emotion; NE, negative emotion. Accomplishment refers to the PERMA dimension and is distinct from CET-4 English achievement.

As shown in Table 4, GLM was positively associated with all observed PERMA subscale scores. The strongest association was observed with meaning (*r_s_* = 0.583, *p* < 0.01), followed by accomplishment (*r_s_* = 0.568), engagement (*r_s_* = 0.542), positive emotion (*r_s_* = 0.540), relationships (*r_s_* = 0.525), and health (r_s_ = 0.482). At the belief level, growth GLB demonstrated the strongest association with overall well-being (*r_s_* = 0.580), followed by growth L2B (*r_s_* = 0.561) and growth ASB (*r_s_* = 0.479). These results indicate a consistent pattern of moderate-to-strong positive associations between growth-oriented language beliefs and observed positive psychological-functioning subscale scores.

In contrast, FLM showed substantially weaker associations with the positive PERMA subscale scores, with coefficients ranging from *r_s_* = 0.157 to 0.203. Its strongest association was with negative emotion (*r_s_* = 0.408, *p* < 0.01). The three fixed-belief dimensions showed a similar pattern, with negative emotion correlating with fixed GLB (*r_s_* = 0.380), fixed L2B (*r_s_* = 0.397), and fixed ASB (*r_s_* = 0.370). Overall, the theory-grounded Growth- and Fixed-oriented composite scores showed different observed association patterns with students’ general psychological well-being.

A complete matrix of Spearman correlations among CET-4 achievement, individual well-being dimensions, and the six language mindset belief dimensions is provided in Supplementary Table S1.

As a revision-stage multiplicity sensitivity analysis, Benjamini–Hochberg false-discovery-rate adjustment did not alter the substantive RQ1 or RQ2 conclusions: all six planned Table 3 correlations and all 64 Table 4 correlations remained significant within their respective families. This broader sensitivity analysis is distinct from the Bonferroni-adjusted *post hoc* comparisons used for RQ4 omnibus tests.

### 4.4 Moderating roles of growth and fixed language mindsets (RQ3)

To address exploratory RQ3, the model entered mean-centered Overall Well-being, GLM, and FLM simultaneously, together with the Well-being × GLM and Well-being × FLM interaction terms. HC3 robust standard errors were used. The model explained 3.0% of the variance in CET-4 scores (*R*^2^ = 0.030; adjusted *R*^2^ = 0.026). Results are presented in Table 5.

**Table 5:** Moderation of the association between psychological well-being and CET-4 achievement by growth and fixed language mindsets.

Term	*B*	Robust SE	95% CI	*p*
Well-being	2.15	2.15	[−2.06, 6.37]	0.317
GLM	9.50	4.26	[1.14, 17.86]	0.026
FLM	−8.23	3.13	[−14.37, −2.08]	0.009
Well-being × GLM	3.02	1.97	[−0.84, 6.87]	0.125
Well-being × FLM	−0.16	1.73	[−3.55, 3.23]	0.927

*N* = 1,030. B, unstandardized regression coefficient; SE, standard error; CI, confidence interval; GLM, growth language mindset; FLM, fixed language mindset. Overall well-being, GLM, and FLM were mean-centered before interaction terms were formed. The theory-grounded GLM and FLM composite scores were entered simultaneously as moderators. Model *R*^2^ = 0.030; adjusted *R*^2^ = 0.026. HC3 heteroscedasticity-robust standard errors are reported.

Neither the well-being × GLM nor the well-being × FLM interaction was statistically significant; the joint robust test of the two interaction terms was also non-significant, *F*(2, 1,024) = 1.24, *p* = 0.290.

Neither interaction term was statistically significant. The centered main-effect estimates were *B* = 2.15 (robust SE = 2.15, 95% CI [−2.06, 6.37], *p =* 0.317) for Overall Well-being, *B* = 9.50 (robust SE = 4.26, 95% CI [1.14, 17.86], *p =* 0.026) for GLM, and *B* = −8.23 (robust SE = 3.13, 95% CI [−14.37, −2.08], *p =* 0.009) for FLM. The well-being × GLM interaction was *B* = 3.02, robust SE = 1.97, 95% CI [−0.84, 6.87], *p =* 0.125, and the well-being × FLM interaction was *B* = −0.16, robust SE = 1.73, 95% CI [−3.55, 3.23], *p =* 0.927. A joint robust test of the two interaction terms was also non-significant, *F*(2, 1,024) = 1.24, *p =* 0.290. Thus, this exploratory analysis did not detect moderation of the well-being–CET-4 association in this sample. It does not establish that language mindsets operate through a different mechanism.

### 4.5 Demographic and learning-history differences (RQ4)

To address RQ4, Mann–Whitney *U* and Kruskal–Wallis *H* tests were conducted to examine differences in language mindsets, psychological well-being, and English achievement across demographic and learning-history groups. Table 6 summarizes the primary results, while Supplementary Table S2 reports the complete set of 119 planned comparisons, including non-significant outcomes and adjusted *post hoc* tests. The primary results below are accompanied by false-discovery-rate sensitivity results where relevant.

**Table 6:** Significant demographic and learning-history differences in psychological well-being, language mindsets, and English achievement.

Characteristic	Outcome	Comparison/Pattern	Test statistic	*p*	Effect size
Gender	CET-4	Female (*Mdn* = 424) > Male (*Mdn* = 400)	*U* = 97,576.5	<0.001	*r* = 0.14
Gender	FLM	Male (*Mdn* = 4.00) > Female (*Mdn* = 3.89)	*U* = 134,506.5	<0.001	*r* = 0.11
Gender	Physical health	Male (*Mdn* = 7.33) > Female (*Mdn* = 7.00)	*U* = 128,235	0.030	*r* = 0.06
Only-child status	CET-4	Only child (*Mdn* = 424) > Non-only child (*Mdn* = 400)	*U* = 113,626.5	<0.001	*r* = 0.12
Hometown locale	CET-4	Urban (*Mdn* = 424), suburban (*Mdn* = 410), rural (*Mdn* = 400); urban > rural in *post hoc* comparison	*H*(2) = 11.729	0.003	*η^2^_H_* = 0.009
Year of study	CET-4	Third (*Mdn* = 439) > first (*Mdn* = 425) > second (*Mdn* = 400); adjusted pairwise results reported in text	*H*(3) = 57.592	<0.001	*η^2^_H_* = 0.053
Future education plans	CET-4	Bachelor (*Mdn* = 429) and top-up (*Mdn* = 426.5) > associate (*Mdn* = 396); adjusted pairwise results reported in text	*H*(2) = 140.080	<0.001	*η^2^_H_* = 0.134
CET-4 attempts	CET-4	Unadjusted omnibus difference; no Bonferroni-adjusted pairwise comparison; FDR sensitivity q = 0.072	*H*(3) = 10.071	0.018	*η^2^_H_* = 0.007

*N* = 1,030. CET-4, College English Test Band 4; FLM, fixed language mindset; GLM, growth language mindset; Mdn, median. For two-group comparisons, effect size is reported as *r*. For Kruskal–Wallis tests, effect size is reported as *η*^2^_H_ = (*H* – *k* + 1)/(*N* – *k*), where *k* is the number of groups. The rural–urban CET-4 comparison remained significant after adjustment for multiple comparisons (adjusted *p* = 0.002). No significant differences in GLM (*p* = 0.061), FLM (*p* = 0.123), or overall psychological well-being (*p* = 0.440) were observed across CET-4-attempt groups.

Gender differences were observed in English achievement, fixed language mindset, and physical health. Female students obtained significantly higher CET-4 scores (*Mdn* = 424) than male students (*Mdn* = 400), *U* = 97,576.5, *p* < 0.001, *r* = 0.14. In contrast, male students reported significantly higher FLM scores (*Mdn* = 4.00) than female students (*Mdn* = 3.89), *U* = 134,506.5, *p* < 0.001, *r* = 0.11. Male students also reported slightly higher physical health scores (*Mdn* = 7.33) than female students (*Mdn* = 7.00), *U* = 128,235, *p =* 0.030, *r* = 0.06. In the 28-test primary RQ4 sensitivity family, the CET-4 and FLM gender differences remained significant after false-discovery-rate adjustment; the Health result did not. The effect sizes were small, particularly for physical health.

A significant difference in CET-4 scores was identified across hometown locales, *H*(2) = 11.729, *p =* 0.003, *η^2^_H_* = 0.009, indicating a very small overall effect. Urban students had the highest median CET-4 score (*Mdn* = 424), followed by suburban (*Mdn* = 410) and rural students (*Mdn* = 400). The adjusted *post hoc* comparison indicated that urban students scored significantly higher than rural students (adjusted *p =* 0.002). This primary result remained significant in the false-discovery-rate sensitivity analysis (q = 0.013).

CET-4 scores also differed across year of study, *H*(3) = 57.592, *p* < 0.001, η*^2^_H_* = 0.053, and future education plans, *H*(2) = 140.080, *p* < 0.001, *η^2^_H_* = 0.134. Bonferroni-adjusted *post hoc* comparisons showed that third-year students (*Mdn* = 439) scored higher than second-year students (*Mdn* = 400) and first-year students (*Mdn* = 425), and first-year students scored higher than second-year students. Students planning bachelor’s study (*Mdn* = 429) or top-up study (*Mdn* = 426.5) scored higher than associate-degree students (*Mdn* = 396). Both omnibus CET-4 results remained significant in the 28-test primary false-discovery-rate sensitivity analysis (*q* < 0.001).

CET-4 scores showed an unadjusted omnibus difference across number-of-attempt groups, *H*(3) = 10.071, *p =* 0.018, *η^2^_H_* = 0.007, indicating a very small effect. However, none of the Bonferroni-adjusted pairwise comparisons reached statistical significance, and the omnibus result did not remain significant in the 28-test primary false-discovery-rate sensitivity analysis (*q* = 0.072). It should therefore be treated as an exploratory signal rather than evidence of a clear ordered achievement difference among individual attempt groups.

No statistically significant cross-sectional differences in GLM (*p =* 0.061), FLM (*p =* 0.123), or Overall Well-being (*p =* 0.440) were observed across number-of-CET-4-attempt groups. These null mindset and well-being comparisons are distinct from the unadjusted CET-4 omnibus result and do not support an inference about within-person change in language mindsets.

## 5 Discussion

### 5.1 Language mindsets and general psychological well-being

The present study extends language mindset research by demonstrating that language-specific beliefs are strongly associated with psychological well-being among Chinese HVSs. The strong relationship between GLM and overall well-being (*r_s_* = 0.589) suggests that growth-oriented language beliefs may represent an important psychological resource in EFL learning contexts.

Rather than functioning only as achievement-related beliefs, language mindsets may shape how learners interpret challenges, maintain engagement, and construct meaning during the learning process. Growth-oriented beliefs may provide learners with an interpretive framework through which language-learning difficulty is perceived as manageable rather than diagnostic of low ability. Such interpretations may be associated with engagement, accomplishment, and meaning, which is consistent with the stronger observed PERMA subscale associations in the present study and also consistent with evidence from Chinese university students showing that growth mindset is associated with emotional well-being partly through perceived stress and psychological resilience (Jia et al., 2022). However, because these mechanisms were not measured in the present study, they should be regarded as directions for future investigation.

At the same time, the weak association between GLM and CET-4 achievement in the present sample (*r_s_* = 0.127) contrasts with stronger associations reported among Chinese students in academically oriented university contexts. Hu et al. (2022), for example, reported a substantially stronger relationship between growth mindset and English-language performance. This contrast suggests that the relationship between growth-oriented beliefs and standardized achievement may vary across educational settings and learner populations. In higher vocational education, CET-4 performance may depend more heavily on prior language preparation, learning opportunities, test preparation, and other contextual factors (Bai, 2020; Ma, 2025), whereas GLM may be more proximally associated with learners’ psychological experiences. Because the present study did not directly compare institutional types, this interpretation requires replication in multisite comparative research.

The exploratory moderation analysis did not detect a Well-being × GLM or Well-being × FLM interaction in this sample. The null interactions should not be interpreted as a theoretical boundary on the role of language mindsets or as evidence that they operate through a different mechanism. Rather, within the precision of the reported confidence intervals and a model explaining little CET-4 variance, the data did not detect conditional associations of the specified form. Future preregistered longitudinal or multisite studies can evaluate this interaction question more decisively.

### 5.2 Growth and fixed language mindsets reflect different psychological patterns

A particularly important finding is that GLM and FLM showed different cross-sectional association patterns with general psychological well-being indicators. GLM was strongly associated with meaning, accomplishment, and engagement, whereas FLM showed its strongest association with negative emotion.

This finding suggests that GLM and FLM should not simply be understood as opposite points on one continuum. Instead, they may represent different psychological orientations influencing how learners experience foreign language learning. This interpretation is consistent with the two-factor structure of the LMI, in which growth and fixed orientations are represented as distinct factors.

The positive, albeit modest, association between GLM and FLM (*r_s_* = 0.244) further suggests that growth- and fixed-oriented beliefs may coexist within individual learners. HVSs may believe that some aspects of language competence are developable while simultaneously endorsing fixed beliefs concerning aptitude or age-related constraints. This pattern supports examining GLM and FLM as related but distinguishable belief orientations rather than collapsing them into a single composite continuum.

### 5.3 The complex role of negative emotions

Negative emotion was positively associated with some positive PERMA indicators in this sample. This observed co-occurrence does not establish that negative emotions benefit psychological functioning. One possible interpretation, informed by prior theory, is that negative emotions may coexist with motivation, self-reflection, or persistence in demanding educational settings. The present data do not test these functions or their cultural origins.

One possible interpretive framework for the present findings is emotional complexity. Cross-cultural research has reported greater co-occurrence of pleasant and unpleasant emotions in some East Asian samples, sometimes linked to dialectical approaches to contradiction rather than a simple positive–negative opposition (Schimmack et al., 2002; Spencer-Rodgers et al., 2010; Grossmann et al., 2016). This literature is consistent with, but does not establish, an explanation for the observed positive correlations involving negative emotion. Because the present study did not measure dialectical thinking, cultural orientation, or other relevant mechanisms, any cultural explanation remains tentative and requires direct empirical testing. Recent evidence also indicates that Chinese thinking style is multidimensional and may relate to mental health through psychological resilience (Huang et al., 2024). Future research should measure culturally patterned thinking styles and related psychological resources directly.

### 5.4 Learner diversity and contextual variation

The demographic findings further illustrate the heterogeneity of Chinese HVSs. Gender was associated with both English achievement and fixed language mindset, although the effect sizes were small. Female students obtained higher CET-4 scores, whereas male students reported slightly stronger fixed language mindset beliefs. A very small gender difference was also identified for physical health. These findings should not be interpreted as evidence of inherent gender differences in language ability or psychological functioning. Rather, they indicate modest group-level variations that may reflect differences in previous learning experiences, engagement patterns, or educational contexts. Therefore, the small gender difference in FLM should be interpreted as a context-specific pattern rather than a stable gender-linked characteristic. Moreover, Lou and Noels (2017) reported no gender differences in language growth mindset, while broader mindset research has produced inconsistent gender patterns across samples (Diseth et al., 2014; Khajavy et al., 2020). Accordingly, the present finding warrants replication before stronger conclusions are drawn about gender and fixed language beliefs among Chinese HVSs.

Furthermore, English achievement varied according to family structure and hometown locale. Only-child students obtained somewhat higher CET-4 scores than students with siblings, while urban students outperformed rural students. Because these effects were small and the present study did not directly measure family resources, school quality, parental investment, or prior English exposure, the observed differences cannot be attributed to any single mechanism. Prior research nevertheless provides a plausible contextual framework for the hometown difference: English language teaching in China has shown regional disparities in English proficiency, previous learning experiences, and access to educational resources, shaped by economic, social, cultural, and pedagogical factors (Hu, 2003; Feng, 2005). These documented disparities are consistent with the possibility that educational opportunity contributes to the observed achievement pattern, but the present data cannot determine whether such factors account for the rural–urban difference because they were not measured directly.

The findings concerning number of CET-4 attempts require particularly cautious interpretation. The unadjusted omnibus Kruskal–Wallis test for CET-4 achievement was significant, but subsequent Bonferroni-adjusted pairwise comparisons were not, and the omnibus result did not remain significant in the primary false-discovery-rate sensitivity analysis. In addition, GLM and FLM showed no statistically significant cross-sectional differences across attempt groups. These are separate findings and do not support an inference that repeated participation shifts learners toward a fixed mindset. Longitudinal research would be required to examine within-person change following repeated success or failure experiences. Achievement appears somewhat more sensitive to students’ demographic and educational backgrounds, whereas the central psychological orientations of GLM and FLM show considerably less demographic differentiation.

### 5.5 Theoretical implications

The present findings offer several theoretical implications for educational psychology and language mindset research. First, in this sample, language mindset scores were more strongly associated with general psychological well-being than with standardized achievement. GLM showed a strong association with Overall Well-being (*r_s_* = 0.589), whereas its association with CET-4 achievement was comparatively weak (*r_s_* = 0.127); Overall Well-being was also weakly associated with CET-4 scores (*r_s_* = 0.110). This cross-sectional pattern may have implications for research that considers language beliefs alongside general psychological functioning. It does not show that PERMA-Profiler scores represent English-learning well-being or that language mindsets produce changes in well-being. Second, the observed PERMA subscale associations provide a more differentiated account of the psychological correlates of growth-oriented beliefs. GLM was particularly strongly associated with meaning (*r_s_* = 0.583), accomplishment (*r_s_* = 0.568), and engagement (*r_s_* = 0.542), while growth-oriented GLB and L2B also showed relatively strong associations with overall well-being. These patterns suggest that the relevance of a growth language mindset may lie not merely in believing that improvement is possible, but in its association with learners’ broader perceptions that language learning is purposeful, engaging, and accompanied by a sense of progress. This pattern is consistent with the language mindset–meaning system, in which beliefs about language ability are embedded in broader processes of interpretation and meaning-making (Lou and Noels, 2019).

Third, the observed association patterns are consistent with treating GLM and FLM as related, theory-grounded orientation composites rather than opposite ends of a single continuum. Although FLM showed only weak associations with the positive PERMA subscale scores, its strongest association was with negative emotion (*r_s_* = 0.408). In contrast, GLM was more strongly associated with the positive psychological-functioning subscale scores. Moreover, GLM and FLM themselves were positively, albeit modestly, associated (*r_s_* = 0.244), indicating that growth- and fixed-oriented beliefs may coexist within the same learner. The current-sample hierarchical CFA provided partial rather than definitive structural support for this interpretation; the composite-score findings should therefore be interpreted cautiously. Fourth, the exploratory moderation analysis did not detect a conditional association between overall psychological well-being and CET-4 achievement as a function of GLM or FLM. This non-detection does not establish a theoretical boundary or an alternative mechanism for language mindsets; it indicates only that the specified interactions were not detected in this cross-sectional sample. Future preregistered longitudinal or multisite studies could test whether such moderation emerges under different educational conditions or at different stages of language learning.

Finally, the findings contribute a strengths-based perspective to research on Chinese HVSs. While English achievement showed modest variation across demographic and educational backgrounds, the core psychological orientations of GLM and FLM displayed considerably less differentiation across some learning-history groups; for example, neither GLM nor FLM differed significantly across CET-4-attempt groups. This distinction suggests that standardized achievement and psychological functioning should not be regarded as interchangeable indicators of learner development. Achievement may reflect a broader combination of educational opportunity, prior preparation, family background, and learning experience, whereas language mindsets and well-being capture different aspects of learners’ psychological engagement with language learning.

### 5.6 Pedagogical implications

The present findings offer several pedagogical implications for English instruction in higher vocational education. First, the results suggest that growth-supportive instructional feedback may be educationally valuable. Rather than framing language performance as evidence of stable ability, instructors may emphasize strategies, revision, effort quality, and progress. For instance, instead of characterizing a weak speaking performance as reflecting low ability, feedback could target developable components such as organization, pronunciation, or rehearsal strategy. Such practices are consistent with a growth-oriented view of language ability without claiming that mindset-oriented feedback will necessarily raise examination scores.

Second, given the strong associations between GLM and meaning, engagement, and accomplishment, instructors may enhance learner motivation by connecting language tasks to HVSs’ future professional identities, workplace communication needs, and everyday communicative goals. Meaning-oriented goal setting can be paired with attainable short-term milestones, such as delivering a brief workplace presentation, completing a mock interview, or improving the organization of a written response, so that students can recognize concrete progress over time.

Third, the findings do not support the assumption that effective language education requires eliminating negative emotional experiences. Negative emotion was associated with both GLM and FLM, though more strongly with the latter. Instructors may consider classroom practices in which difficulty, anxiety, and mistakes are acknowledged without being interpreted as evidence of fixed inability. Error-normalizing practices, low-stakes rehearsal opportunities, post-task reflection, and feedback that distinguishes temporary performance from underlying capacity are pedagogical possibilities rather than empirically demonstrated effects of this study.

Fourth, the demographic findings point to the importance of context-sensitive rather than deficit-oriented support. Although small achievement differences were observed by gender, only-child status, and hometown locale, these results should not be used to label particular groups as inherently weaker learners. Instead, they suggest that instructors and institutions should attend to differences in prior educational opportunities and access to language-learning resources. Diagnostic support, differentiated learning opportunities, accessible extracurricular resources, and additional academic guidance may be more appropriate responses than treating demographic membership as an explanation for achievement differences.

Taken together, the pedagogical implication is not that instructors should pursue general psychological well-being or GLM primarily as instruments for raising CET-4 scores. Instead, the cross-sectional associations may have implications for learning environments that acknowledge development, meaning, engagement, manageable challenge, and accomplishment. This is a pedagogical interpretation, not evidence that these practices change well-being, mindsets, or achievement.

### 5.7 Limitations and future research

Several limitations should be considered.

First, the cross-sectional correlational design precludes causal inferences. Although associations were identified among language mindsets, general psychological well-being, and English achievement, their temporal direction remains undetermined. For instance, GLM and well-being may be reciprocally associated, or both may relate to unmeasured contextual factors. The mechanisms discussed above are theoretical possibilities rather than demonstrated causal processes.

Second, the sample was recruited from a single private vocational and technical university in Shanghai. Despite the relatively large sample size (*N* = 1,030), higher vocational education in China encompasses considerable institutional, regional, and socioeconomic diversity. Therefore, the findings should not be generalized automatically to public vocational colleges or institutions in other regions. Future research should employ multi-site sampling across diverse institutional types and geographic regions to examine the robustness of the observed mindset–well-being patterns.

Third, CET-4 achievement was assessed via self-reported scores rather than official records, introducing the potential for recall error or response bias. Although scores outside the admissible range (220–710) were excluded, this criterion resulted in the exclusion of nearly half the consenting respondents (*n* = 928). While restricting the analysis to the 1,030 valid cases ensured a consistent analytical sample, it may have introduced selection bias if excluded respondents differed systematically from those retained. Future studies should, where feasible, obtain verified achievement data and examine whether participants with and without available CET-4 results differ in relevant psychological or demographic characteristics.

Fourth, general psychological well-being and language mindsets were measured exclusively through self-report questionnaires. The PERMA-Profiler does not assess EFL-specific well-being or classroom-specific emotion, so the results cannot be taken as a direct account of students’ English-learning well-being. Although the PERMA-Profiler and LMI demonstrated strong internal consistency, self-report measures cannot fully capture the behavioral and contextual processes through which learners respond to challenges. Future studies could combine questionnaires with classroom observations, interviews, learning-behavior indicators, EFL-specific well-being measures, or repeated experience-sampling measures.

Fifth, the study identified associations but did not directly test the psychological mechanisms connecting language mindsets, general psychological well-being, and achievement. The strong associations of GLM with meaning, accomplishment, and engagement suggest theoretically relevant pathways, such as resilience, self-regulation, or emotion regulation, that warrant direct examination. Conversely, the stronger association between FLM and negative emotion suggests a possible emotional pathway. These mechanisms should be tested prospectively rather than inferred from concurrent correlations.

Finally, several statistically significant associations with CET-4 achievement and demographic characteristics were small in magnitude. Given the large sample size, statistical power was sufficient to detect small effects; statistical significance should not be equated with educational importance. In this sample, language mindset scores had stronger associations with general psychological well-being than with standardized achievement. Future studies should incorporate variables such as prior English proficiency, family socioeconomic resources, and school quality to clarify these contextual patterns.

## 6 Conclusion

This study investigated the associations among psychological well-being, language mindsets, and English achievement among Chinese higher vocational students. The findings demonstrated that growth language mindset was strongly associated with psychological well-being, particularly the observed PERMA subscale scores for meaning, accomplishment, and engagement. Meanwhile, associations between psychological variables and CET-4 achievement were statistically significant but relatively small, suggesting that language beliefs and well-being may be more closely related to learners’ psychological experiences than to standardized examination outcomes.

The observed association patterns for the two theory-grounded LMI composite scores differed: growth-oriented beliefs were consistently associated with positive PERMA subscale scores, whereas fixed language mindset showed stronger relationships with negative emotional experiences. Because the PERMA-Profiler assesses general psychological well-being, these associations should not be treated as direct evidence about EFL-specific well-being or classroom emotion. Current-sample structural evidence for the LMI composite scores was partial rather than definitive. The co-occurrence of negative emotion with positive psychological indicators may reflect complexity in learners’ reported experiences, but cultural explanations remain speculative because culture was not measured. Neither GLM nor FLM significantly moderated the association between Overall Well-being and CET-4 achievement; these mindset composite scores did not detectably change the strength of that association in this sample. Demographic analyses further demonstrated that English achievement varied modestly across learner backgrounds. Female students, only-child students, and students from urban areas obtained higher CET-4 scores than their respective comparison groups, while male students reported slightly stronger fixed language mindset beliefs. However, these differences were generally small in magnitude. Furthermore, neither GLM nor FLM differed significantly across CET-4-attempt groups; the cross-sectional data do not support interpreting number of attempts as a determinant of language mindset. Taken together, the findings show associations between general psychological well-being and language mindsets, whereas standardized English achievement may reflect a broader combination of psychological, educational, and contextual influences. Overall, this study contributes to educational psychology by examining language mindsets as psychologically meaningful beliefs, not solely as achievement-related factors. By focusing on higher vocational students, it provides a strengths-based perspective on an underrepresented learner population and identifies associations that may be relevant to future work on students’ psychological resources, sense of meaning, and language beliefs.

## Data Availability

The raw data supporting the conclusions of this article will be made available by the authors, without undue reservation.
