# Supplementary material for: Unlocking potential: language mindsets, psychological well-being, and English achievement among Chinese higher vocational students
Source: Front Psychol. 2026 Oct 5;17:1969912. doi: 10.3389/fpsyg.2026.1969912 (PMC13677826; doi:10.3389/fpsyg.2026.1969912)
Supplement: Supplementary file 1 [file Supplementary_file_1.DOCX]

Supplementary Material

# 1 Supplementary Table S1

*Full Spearman Correlation Matrix Among English Achievement, Well-Being Dimensions, and Language Mindset Beliefs*

| **Variable** | **1** | **2** | **3** | **4** | **5** | **6** | **7** | **8** | **9** | **10** | **11** | **12** | **13** | **14** |
| --- | --- | --- | --- | --- | --- | --- | --- | --- | --- | --- | --- | --- | --- | --- |
| 1. CET-4 | — |  |  |  |  |  |  |  |  |  |  |  |  |  |
| 2. Positive emotion | .094** | — |  |  |  |  |  |  |  |  |  |  |  |  |
| 3. Engagement | .129** | .831** | — |  |  |  |  |  |  |  |  |  |  |  |
| 4. Relationships | .063** | .830** | .780** | — |  |  |  |  |  |  |  |  |  |  |
| 5. Meaning | .115** | .837** | .815** | .775** | — |  |  |  |  |  |  |  |  |  |
| 6. Accomplishment | .125** | .818** | .834** | .773** | .906** | — |  |  |  |  |  |  |  |  |
| 7. Health | .049 | .807** | .698** | .735** | .755** | .749** | — |  |  |  |  |  |  |  |
| 8. Negative emotion | .019 | .194** | .353** | .269** | .303** | .352** | .201** | — |  |  |  |  |  |  |
| 9. Fixed GLB | −.048 | .157** | .182** | .162** | .174** | .187** | .160** | .380** | — |  |  |  |  |  |
| 10. Growth GLB | .091** | .531** | .531** | .502** | .572** | .580** | .503** | .222** | .299** | — |  |  |  |  |
| 11. Fixed L2B | −.112** | .196** | .245** | .217** | .198** | .217** | .193** | .397** | .713** | .271** | — |  |  |  |
| 12. Growth L2B | .166** | .510** | .532** | .493** | .563** | .542** | .463** | .249** | .246** | .725** | .234** | — |  |  |
| 13. Fixed ASB | −.049 | .163** | .212** | .154** | .187** | .204** | .168** | .370** | .688** | .290** | .739** | .248** | — |  |
| 14. Growth ASB | .082** | .454** | .443** | .448** | .469** | .442** | .374** | .243** | .211** | .603** | .232** | .727** | .180** | — |

*Note. N = 1,030. Values are Spearman rank-order correlation coefficients (rs). CET-4 = College English Test Band 4; GLB = general language intelligence beliefs; L2B = second-language aptitude beliefs; ASB = age-sensitivity beliefs. **p < .01 (two-tailed). The absence of a significance marker indicates that the correlation was not statistically significant. Accomplishment refers to the PERMA dimension and should not be confused with CET-4 English achievement.*

# 2 Supplementary Table S2

*Complete RQ4 Mann–Whitney U and Kruskal–Wallis H Comparison Matrix*

*Note. N = 1,030. The matrix crosses the 17 outcomes reported in Table 2 with the seven planned demographic and learning-history grouping variables (119 omnibus comparisons). Effect size is r for Mann–Whitney U tests and η²_H_ for Kruskal–Wallis H tests. q values are Benjamini–Hochberg false-discovery-rate sensitivity values across all 119 omnibus comparisons. Pairwise comparisons were Bonferroni-adjusted within raw-significant Kruskal–Wallis tests; significant adjusted pairwise comparisons are reported after the matrix.*

| Grouping variable | Outcome | Test | Statistic | df | p | Effect | FDR q (119) |
| --- | --- | --- | --- | --- | --- | --- | --- |
| Gender | CET-4 | Mann–Whitney U | 139424.500 |  | < .001 | 0.145 r | < .001 |
| Gender | Overall well-being | Mann–Whitney U | 116418.500 |  | 0.644 | 0.014 r | 0.898 |
| Gender | Positive emotion | Mann–Whitney U | 118457.000 |  | 0.992 | 0.000 r | 0.992 |
| Gender | Engagement | Mann–Whitney U | 111373.500 |  | 0.113 | 0.049 r | 0.561 |
| Gender | Relationships | Mann–Whitney U | 119973.500 |  | 0.743 | 0.010 r | 0.918 |
| Gender | Meaning | Mann–Whitney U | 112210.000 |  | 0.162 | 0.044 r | 0.624 |
| Gender | Accomplishment | Mann–Whitney U | 113175.500 |  | 0.237 | 0.037 r | 0.674 |
| Gender | Health | Mann–Whitney U | 108766.000 |  | 0.030 | 0.067 r | 0.284 |
| Gender | Negative emotion | Mann–Whitney U | 115645.500 |  | 0.526 | 0.020 r | 0.824 |
| Gender | FLM | Mann–Whitney U | 102494.500 |  | < .001 | 0.111 r | 0.007 |
| Gender | Fixed GLB | Mann–Whitney U | 108681.500 |  | 0.028 | 0.068 r | 0.284 |
| Gender | Fixed L2B | Mann–Whitney U | 103177.000 |  | < .001 | 0.107 r | 0.010 |
| Gender | Fixed ASB | Mann–Whitney U | 98448.000 |  | < .001 | 0.140 r | < .001 |
| Gender | GLM | Mann–Whitney U | 119244.500 |  | 0.869 | 0.005 r | 0.966 |
| Gender | Growth GLB | Mann–Whitney U | 114666.000 |  | 0.387 | 0.027 r | 0.792 |
| Gender | Growth L2B | Mann–Whitney U | 114976.500 |  | 0.426 | 0.025 r | 0.815 |
| Gender | Growth ASB | Mann–Whitney U | 124983.000 |  | 0.141 | 0.046 r | 0.608 |
| Hometown locale | CET-4 | Kruskal–Wallis H | 11.729 | 2 | 0.003 | 0.009 eta2H | 0.042 |
| Hometown locale | Overall well-being | Kruskal–Wallis H | 3.874 | 2 | 0.144 | 0.002 eta2H | 0.608 |
| Hometown locale | Positive emotion | Kruskal–Wallis H | 4.002 | 2 | 0.135 | 0.002 eta2H | 0.608 |
| Hometown locale | Engagement | Kruskal–Wallis H | 5.637 | 2 | 0.060 | 0.004 eta2H | 0.429 |
| Hometown locale | Relationships | Kruskal–Wallis H | 3.388 | 2 | 0.184 | 0.001 eta2H | 0.663 |
| Hometown locale | Meaning | Kruskal–Wallis H | 3.192 | 2 | 0.203 | 0.001 eta2H | 0.664 |
| Hometown locale | Accomplishment | Kruskal–Wallis H | 2.713 | 2 | 0.258 | 0.001 eta2H | 0.674 |
| Hometown locale | Health | Kruskal–Wallis H | 1.026 | 2 | 0.599 | -0.001 eta2H | 0.880 |
| Hometown locale | Negative emotion | Kruskal–Wallis H | 1.486 | 2 | 0.476 | -0.001 eta2H | 0.815 |
| Hometown locale | FLM | Kruskal–Wallis H | 0.597 | 2 | 0.742 | -0.001 eta2H | 0.918 |
| Hometown locale | Fixed GLB | Kruskal–Wallis H | 0.951 | 2 | 0.622 | -0.001 eta2H | 0.896 |
| Hometown locale | Fixed L2B | Kruskal–Wallis H | 0.254 | 2 | 0.881 | -0.002 eta2H | 0.966 |
| Hometown locale | Fixed ASB | Kruskal–Wallis H | 0.783 | 2 | 0.676 | -0.001 eta2H | 0.898 |
| Hometown locale | GLM | Kruskal–Wallis H | 1.385 | 2 | 0.500 | -0.001 eta2H | 0.824 |
| Hometown locale | Growth GLB | Kruskal–Wallis H | 0.090 | 2 | 0.956 | -0.002 eta2H | 0.979 |
| Hometown locale | Growth L2B | Kruskal–Wallis H | 3.417 | 2 | 0.181 | 0.001 eta2H | 0.663 |
| Hometown locale | Growth ASB | Kruskal–Wallis H | 1.338 | 2 | 0.512 | -0.001 eta2H | 0.824 |
| Only-child status | CET-4 | Mann–Whitney U | 149749.500 |  | < .001 | 0.118 r | 0.003 |
| Only-child status | Overall well-being | Mann–Whitney U | 134014.500 |  | 0.625 | 0.015 r | 0.896 |
| Only-child status | Positive emotion | Mann–Whitney U | 131922.000 |  | 0.961 | 0.002 r | 0.979 |
| Only-child status | Engagement | Mann–Whitney U | 133265.000 |  | 0.740 | 0.010 r | 0.918 |
| Only-child status | Relationships | Mann–Whitney U | 132012.000 |  | 0.946 | 0.002 r | 0.979 |
| Only-child status | Meaning | Mann–Whitney U | 135235.500 |  | 0.454 | 0.023 r | 0.815 |
| Only-child status | Accomplishment | Mann–Whitney U | 135666.000 |  | 0.402 | 0.026 r | 0.797 |
| Only-child status | Health | Mann–Whitney U | 125909.000 |  | 0.223 | 0.038 r | 0.664 |
| Only-child status | Negative emotion | Mann–Whitney U | 140938.500 |  | 0.051 | 0.061 r | 0.407 |
| Only-child status | FLM | Mann–Whitney U | 136950.500 |  | 0.267 | 0.035 r | 0.674 |
| Only-child status | Fixed GLB | Mann–Whitney U | 136825.000 |  | 0.277 | 0.034 r | 0.674 |
| Only-child status | Fixed L2B | Mann–Whitney U | 135171.500 |  | 0.459 | 0.023 r | 0.815 |
| Only-child status | Fixed ASB | Mann–Whitney U | 135716.500 |  | 0.393 | 0.027 r | 0.792 |
| Only-child status | GLM | Mann–Whitney U | 130937.000 |  | 0.874 | 0.005 r | 0.966 |
| Only-child status | Growth GLB | Mann–Whitney U | 129151.000 |  | 0.587 | 0.017 r | 0.873 |
| Only-child status | Growth L2B | Mann–Whitney U | 133083.000 |  | 0.765 | 0.009 r | 0.918 |
| Only-child status | Growth ASB | Mann–Whitney U | 129661.500 |  | 0.663 | 0.014 r | 0.898 |
| Year of study | CET-4 | Kruskal–Wallis H | 57.592 | 3 | < .001 | 0.053 eta2H | < .001 |
| Year of study | Overall well-being | Kruskal–Wallis H | 2.298 | 3 | 0.513 | -0.001 eta2H | 0.824 |
| Year of study | Positive emotion | Kruskal–Wallis H | 1.713 | 3 | 0.634 | -0.001 eta2H | 0.898 |
| Year of study | Engagement | Kruskal–Wallis H | 6.346 | 3 | 0.096 | 0.003 eta2H | 0.504 |
| Year of study | Relationships | Kruskal–Wallis H | 0.282 | 3 | 0.963 | -0.003 eta2H | 0.979 |
| Year of study | Meaning | Kruskal–Wallis H | 3.594 | 3 | 0.309 | 0.001 eta2H | 0.703 |
| Year of study | Accomplishment | Kruskal–Wallis H | 5.345 | 3 | 0.148 | 0.002 eta2H | 0.608 |
| Year of study | Health | Kruskal–Wallis H | 2.023 | 3 | 0.568 | -0.001 eta2H | 0.865 |
| Year of study | Negative emotion | Kruskal–Wallis H | 6.321 | 3 | 0.097 | 0.003 eta2H | 0.504 |
| Year of study | FLM | Kruskal–Wallis H | 0.542 | 3 | 0.910 | -0.002 eta2H | 0.966 |
| Year of study | Fixed GLB | Kruskal–Wallis H | 2.666 | 3 | 0.446 | -0.000 eta2H | 0.815 |
| Year of study | Fixed L2B | Kruskal–Wallis H | 1.060 | 3 | 0.787 | -0.002 eta2H | 0.918 |
| Year of study | Fixed ASB | Kruskal–Wallis H | 0.843 | 3 | 0.839 | -0.002 eta2H | 0.951 |
| Year of study | GLM | Kruskal–Wallis H | 6.312 | 3 | 0.097 | 0.003 eta2H | 0.504 |
| Year of study | Growth GLB | Kruskal–Wallis H | 8.048 | 3 | 0.045 | 0.005 eta2H | 0.383 |
| Year of study | Growth L2B | Kruskal–Wallis H | 6.320 | 3 | 0.097 | 0.003 eta2H | 0.504 |
| Year of study | Growth ASB | Kruskal–Wallis H | 1.514 | 3 | 0.679 | -0.001 eta2H | 0.898 |
| Accommodation | CET-4 | Mann–Whitney U | 20836.000 |  | 0.574 | 0.018 r | 0.865 |
| Accommodation | Overall well-being | Mann–Whitney U | 20325.500 |  | 0.776 | 0.009 r | 0.918 |
| Accommodation | Positive emotion | Mann–Whitney U | 19338.500 |  | 0.802 | 0.008 r | 0.922 |
| Accommodation | Engagement | Mann–Whitney U | 20973.000 |  | 0.524 | 0.020 r | 0.824 |
| Accommodation | Relationships | Mann–Whitney U | 20331.500 |  | 0.773 | 0.009 r | 0.918 |
| Accommodation | Meaning | Mann–Whitney U | 20491.000 |  | 0.707 | 0.012 r | 0.915 |
| Accommodation | Accomplishment | Mann–Whitney U | 20025.500 |  | 0.903 | 0.004 r | 0.966 |
| Accommodation | Health | Mann–Whitney U | 19731.000 |  | 0.970 | 0.001 r | 0.979 |
| Accommodation | Negative emotion | Mann–Whitney U | 19346.500 |  | 0.806 | 0.008 r | 0.922 |
| Accommodation | FLM | Mann–Whitney U | 21715.000 |  | 0.298 | 0.032 r | 0.695 |
| Accommodation | Fixed GLB | Mann–Whitney U | 21275.500 |  | 0.421 | 0.025 r | 0.815 |
| Accommodation | Fixed L2B | Mann–Whitney U | 21619.000 |  | 0.319 | 0.031 r | 0.703 |
| Accommodation | Fixed ASB | Mann–Whitney U | 21472.000 |  | 0.360 | 0.029 r | 0.765 |
| Accommodation | GLM | Mann–Whitney U | 19654.000 |  | 0.937 | 0.002 r | 0.979 |
| Accommodation | Growth GLB | Mann–Whitney U | 20300.000 |  | 0.783 | 0.009 r | 0.918 |
| Accommodation | Growth L2B | Mann–Whitney U | 20026.500 |  | 0.901 | 0.004 r | 0.966 |
| Accommodation | Growth ASB | Mann–Whitney U | 19572.000 |  | 0.900 | 0.004 r | 0.966 |
| Future education plans | CET-4 | Kruskal–Wallis H | 140.080 | 2 | < .001 | 0.134 eta2H | < .001 |
| Future education plans | Overall well-being | Kruskal–Wallis H | 2.522 | 2 | 0.283 | 0.001 eta2H | 0.674 |
| Future education plans | Positive emotion | Kruskal–Wallis H | 2.777 | 2 | 0.249 | 0.001 eta2H | 0.674 |
| Future education plans | Engagement | Kruskal–Wallis H | 3.633 | 2 | 0.163 | 0.002 eta2H | 0.624 |
| Future education plans | Relationships | Kruskal–Wallis H | 2.107 | 2 | 0.349 | 0.000 eta2H | 0.755 |
| Future education plans | Meaning | Kruskal–Wallis H | 3.115 | 2 | 0.211 | 0.001 eta2H | 0.664 |
| Future education plans | Accomplishment | Kruskal–Wallis H | 2.999 | 2 | 0.223 | 0.001 eta2H | 0.664 |
| Future education plans | Health | Kruskal–Wallis H | 0.789 | 2 | 0.674 | -0.001 eta2H | 0.898 |
| Future education plans | Negative emotion | Kruskal–Wallis H | 1.883 | 2 | 0.390 | -0.000 eta2H | 0.792 |
| Future education plans | FLM | Kruskal–Wallis H | 5.060 | 2 | 0.080 | 0.003 eta2H | 0.499 |
| Future education plans | Fixed GLB | Kruskal–Wallis H | 6.949 | 2 | 0.031 | 0.005 eta2H | 0.284 |
| Future education plans | Fixed L2B | Kruskal–Wallis H | 2.742 | 2 | 0.254 | 0.001 eta2H | 0.674 |
| Future education plans | Fixed ASB | Kruskal–Wallis H | 3.316 | 2 | 0.191 | 0.001 eta2H | 0.664 |
| Future education plans | GLM | Kruskal–Wallis H | 1.166 | 2 | 0.558 | -0.001 eta2H | 0.863 |
| Future education plans | Growth GLB | Kruskal–Wallis H | 2.560 | 2 | 0.278 | 0.001 eta2H | 0.674 |
| Future education plans | Growth L2B | Kruskal–Wallis H | 2.672 | 2 | 0.263 | 0.001 eta2H | 0.674 |
| Future education plans | Growth ASB | Kruskal–Wallis H | 0.550 | 2 | 0.759 | -0.001 eta2H | 0.918 |
| Number of CET-4 attempts | CET-4 | Kruskal–Wallis H | 10.071 | 3 | 0.018 | 0.007 eta2H | 0.214 |
| Number of CET-4 attempts | Overall well-being | Kruskal–Wallis H | 2.699 | 3 | 0.440 | -0.000 eta2H | 0.815 |
| Number of CET-4 attempts | Positive emotion | Kruskal–Wallis H | 2.278 | 3 | 0.517 | -0.001 eta2H | 0.824 |
| Number of CET-4 attempts | Engagement | Kruskal–Wallis H | 2.520 | 3 | 0.472 | -0.000 eta2H | 0.815 |
| Number of CET-4 attempts | Relationships | Kruskal–Wallis H | 1.519 | 3 | 0.678 | -0.001 eta2H | 0.898 |
| Number of CET-4 attempts | Meaning | Kruskal–Wallis H | 2.479 | 3 | 0.479 | -0.001 eta2H | 0.815 |
| Number of CET-4 attempts | Accomplishment | Kruskal–Wallis H | 1.466 | 3 | 0.690 | -0.001 eta2H | 0.903 |
| Number of CET-4 attempts | Health | Kruskal–Wallis H | 1.160 | 3 | 0.763 | -0.002 eta2H | 0.918 |
| Number of CET-4 attempts | Negative emotion | Kruskal–Wallis H | 2.642 | 3 | 0.450 | -0.000 eta2H | 0.815 |
| Number of CET-4 attempts | FLM | Kruskal–Wallis H | 5.777 | 3 | 0.123 | 0.003 eta2H | 0.585 |
| Number of CET-4 attempts | Fixed GLB | Kruskal–Wallis H | 4.502 | 3 | 0.212 | 0.001 eta2H | 0.664 |
| Number of CET-4 attempts | Fixed L2B | Kruskal–Wallis H | 11.717 | 3 | 0.008 | 0.008 eta2H | 0.111 |
| Number of CET-4 attempts | Fixed ASB | Kruskal–Wallis H | 3.946 | 3 | 0.267 | 0.001 eta2H | 0.674 |
| Number of CET-4 attempts | GLM | Kruskal–Wallis H | 7.361 | 3 | 0.061 | 0.004 eta2H | 0.429 |
| Number of CET-4 attempts | Growth GLB | Kruskal–Wallis H | 3.561 | 3 | 0.313 | 0.001 eta2H | 0.703 |
| Number of CET-4 attempts | Growth L2B | Kruskal–Wallis H | 7.005 | 3 | 0.072 | 0.004 eta2H | 0.474 |
| Number of CET-4 attempts | Growth ASB | Kruskal–Wallis H | 4.499 | 3 | 0.212 | 0.001 eta2H | 0.664 |

*Significant Bonferroni-adjusted post-hoc comparisons from raw-significant omnibus tests*

| Grouping variable | Outcome | Comparison | U | raw p | Bonferroni-adjusted p |
| --- | --- | --- | --- | --- | --- |
| Hometown locale | CET-4 | Rural vs. Urban | 54457.0 | < .001 | 0.002 |
| Year of study | CET-4 | Second vs. Third | 17530.5 | < .001 | < .001 |
| Year of study | CET-4 | Second vs. First | 79453.5 | < .001 | < .001 |
| Year of study | CET-4 | Third vs. First | 21430.0 | 0.002 | 0.011 |
| Future education plans | CET-4 | Associate vs. Top-up | 9308.0 | < .001 | < .001 |
| Future education plans | CET-4 | Associate vs. Bachelor | 67020.5 | < .001 | < .001 |
| Future education plans | Fixed GLB | Associate vs. Bachelor | 107349.0 | 0.009 | 0.026 |
| Number of CET-4 attempts | Fixed L2B | 2 vs. 3 | 4144.5 | 0.006 | 0.035 |
| Number of CET-4 attempts | Fixed L2B | 1 vs. 3 | 13226.0 | 0.003 | 0.021 |

*For CET-4 attempts, the unadjusted CET-4 omnibus result was H(3) = 10.071, p = .018, but no Bonferroni-adjusted pairwise CET-4 comparison was significant; the 28-test primary-outcome FDR sensitivity value was q = .072.*
